# Comparative Analysis of Pathology Foundation Models for Automated Detection of Tertiary Lymphoid Structures in Hematoxylin-and-Eosin-Stained Digital Pathology Images

**DOI:** 10.34133/csbj.0086

**Published:** 2026-05-14

**Authors:** Meijian Guan, Yu Sun, Merzu Belete, Anantharaman Muthuswamy, Maximilian Farma, Jenny Kaufmann, Mirna Lechpammer, Sriram Sridhar, Brandon W. Higgs, Han Si

**Affiliations:** ^1^ Translational Data Science, Genmab, Princeton, NJ, USA.; ^2^ Pathology, Genmab, Princeton, NJ, USA.; ^3^ Cornell University, Ithaca, NY, USA.; ^4^ Harvard University, Cambridge, MA, USA.

## Abstract

Tertiary lymphoid structures (TLSs) are observed in solid tumors and are associated with improved immunotherapy outcomes, yet their dynamic nature makes TLS identification in clinical samples challenging. Pathology foundation models have recently emerged as powerful tools in computational pathology. In this study, we developed a computational approach to identifying TLSs across cancer types using pathology foundation models, with ImageNet-pretrained ResNet50 as a baseline. Models were applied to hematoxylin and eosin images from pancreatic ductal adenocarcinoma (PDAC) and head and neck squamous cell carcinoma (HNSCC) from The Cancer Genome Atlas and a licensed Real-World Evidence cohort. TLS status was evaluated using both pathologist annotations and transcriptomic-signature-based labels. Pathologist-identified TLS-positive tumors showed higher TLS signature expression in both diseases. Among the models assessed, PLIP and CTransPath demonstrated strong performance in PDAC (area under the curve = 0.94 and 0.89), whereas all models struggled in HNSCC, likely reflecting greater tumor microenvironment heterogeneity. Despite effectively detecting TLSs in PDAC, foundation models performed poorly when predicting transcriptomic-signature-based outcomes in both PDAC and HNSCC. This discrepancy suggests that transcriptomic TLS signatures may capture broader or more transient biological processes, while pathology-based assessment reflects visible TLS morphology, more closely aligned with the features foundation models learn. Overall, these findings highlight the potential of pathology foundation models for TLS detection and immune microenvironment profiling using routinely collected biosamples. However, further refinement is needed to improve performance in tumors with complex tumor microenvironment and to enable reliable prediction of transcriptomic biomarkers directly from hematoxylin and eosin slides.

## Graphical Abstract

**Figure:**
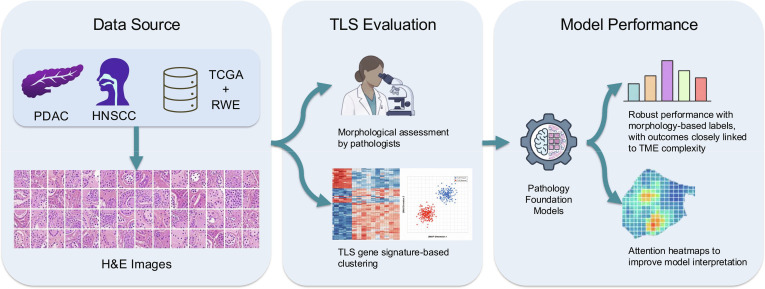

## Introduction

Tertiary lymphoid structures (TLSs) [1] are ectopic lymphoid aggregate structures of immune cells that form within nonlymphoid tissues, including in the tumor microenvironment (TME) [2]. Found in various cancers, including head and neck squamous cell carcinoma (HNSCC) [3] and pancreatic ductal adenocarcinoma (PDAC) [4,5], TLSs are composed of B cell follicles, T cell zones, and dendritic cells and function as sites for local immune activation.

TLSs are crucial in cancer immunotherapy due to their role in modulating antitumor immune responses [6–8]. They act as hubs for antigen presentation, T cell activation, and B cell maturation, fostering a robust immune response against tumor cells. For example, TLS presence is associated with better response rates to immune checkpoint inhibitors, as they provide a localized immune environment that enhances T cell activation and cytotoxic activity. Consequently, TLSs are emerging as predictive biomarkers for immunotherapy efficacy and overall survival in patients with cancer.

Recent studies have highlighted the clinical significance of TLSs in various cancers. A meta-analysis encompassing 17 studies with 4,291 patients with lung cancer revealed that a high TLS presence correlates with improved overall survival (hazard ratio [HR] = 0.66, 95% confidence interval [CI]: 0.50 to 0.88) and disease-free survival (HR = 0.46, 95% CI: 0.33 to 0.64) [9]. Similarly, research focusing on digestive system cancers demonstrated that the absence of TLSs is associated with poorer overall survival (HR = 1.74, 95% CI: 1.50 to 2.03) and recurrence-free survival (HR = 1.96, 95% CI: 1.58 to 2.44) [10]. Furthermore, the presence of mature TLSs has been linked to enhanced responses to immune checkpoint inhibitors, suggesting their potential as predictive biomarkers for immunotherapy efficacy [11]. In HNSCC, intratumoral TLSs have been associated with improved patient survival and better responses to immunotherapy [12]. Similarly, research indicates that patients with PDAC who exhibit TLSs tend to have a more favorable prognosis compared to those who do not [5,13,14]. Collectively, these findings highlight the prognostic and therapeutic relevance of TLSs across multiple cancer types.

TLS detection has traditionally relied on RNA sequencing (RNA-seq) to identify specific gene signatures [3,15,16], manual histology-based assessment to visually identify structures within tumor tissues [17,18], or the detection of specific TLS-associated markers (e.g., CD3, CD20, CD23, and DC-LAMP) by immunohistochemistry [6,18–20]. While informative, these methods are time-consuming, lack scalability, and often miss spatial context.

**Fig. 1.**
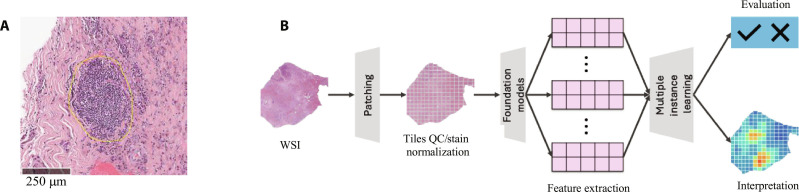
(A) Illustrative tertiary lymphoid structures (TLSs) in a hematoxylin and eosin (H&E) slide marked by a pathologist showing as an organized lymphoid aggregate (follicle-like) typically rounded/ovoid and spatially localized within the tumor microenvironment. (B) High-level workflow for the histopathology image processing.

Recent advancements in digital pathology and artificial intelligence (AI) have enabled the use of deep learning algorithms to detect TLSs (Fig. 1A) directly from hematoxylin and eosin (H&E) whole-slide images (WSIs) [21]. Published models leveraging convolutional neural networks (CNNs) have demonstrated high accuracy and scalability in automating TLS identification [22–24]. Despite these developments, the use of different emerging foundation models, which are pretrained architectures using large amount of histology images, remains underexplored in detecting TLSs. Foundation models offer potential advantages by leveraging excellent transfer learning, enabling feature extraction from diverse datasets prior to a variety of downstream tasks such as tumor detection, biomarker prediction, and survival analysis. Several pathology foundation models use self-supervised learning to enhance image analysis. CTransPath (Swin Transformer) [25], Histossl (iBOT) [26], Virchow (DINOv2) [27], PLIP (Contrastive Learning) [28], and RETCCL (Clustering-guided Contrastive Learning) [29] specialize in tasks such as biomarker classification and image retrieval, demonstrating marked advancements in histopathology.

In this study (Fig. 1B), H&E WSIs were embedded using pretrained pathology foundation models and aggregated with attention-based multiple instance learning to predict TLS status. Predictions were evaluated against pathologist-defined TLS labels and transcriptomic TLS groupings, with attention maps used to illustrate representative regions driving model outputs. We benchmarked the performance of several commonly used pathology foundation models, including PLIP and Virchow against the pretrained model ResNet50, in identifying TLSs within PDAC and HNSCC samples from The Cancer Genome Atlas (TCGA) and Real-World Evidence (RWE) datasets.

By leveraging the strengths of these foundation models in histopathological image analysis, we aim to establish a robust methodology for objective and automated TLS identification and further assess its utility of being a prognostic biomarker associated with clinical outcomes in PDAC and HNSCC.

## Methods

### TCGA PDAC and HNSCC cohorts

TCGA provides comprehensive genomic profiles for various cancer types, including PDAC and HNSCC. We used 163 patients with WSIs (20× magnification) and 173 patients with RNA-seq data, as well as their corresponding clinical information, from a diverse set of patients with pancreatic cancer (Table 1). Similarly, we selected a subset of the TCGA HNSCC cohort, consisting of detailed clinical information, histopathological images (*N* = 100), and transcriptomic data (*N* = 93) from patients with head and neck cancer (Table 2). In addition, this study also utilized an HNSCC cohort from a licensed RWE cohort (Tempus AI Inc.; Table 2), which comprises longitudinal data from geographically diverse oncology practices. The dataset included 188 samples profiled with histopathology images, of which 185 had matched whole-transcriptome RNA-seq, as previously described [30].

**Table 1.** Patient characteristics of PDAC-TCGA cohort

PDAC-TCGA		RNA-seq-based patient group		Pathology-based patient group	
		TLS-high	TLS-low	TLS-present	TLS-absent
Total		84	89	91	72
Gender (%F)		47 (56%)	31 (35%)	43 (47%)	30 (42%)
Mean of age (SD)		64.9 (10.9)	64.5 (10.9)	64.5 (11.6)	65.3 (10.6)
Pathological stage	Stage I	9	12	12	8
	Stage II	71	72	74	59
	Stage III+	4	3	4	4
	Missing	0	0	1	0

TLS, tertiary lymphoid structure; PDAC, pancreatic ductal adenocarcinoma; TCGA, The Cancer Genome Atlas; RNA-seq, RNA sequencing; F, female.

**Table 2.** Patient characteristics of HNSCC-TCGA and HNSCC-RWE cohorts

		HNSCC-TCGA				HNSCC-RWE			
HNSCC		RNA-seq-based patient group		Pathology-based patient group		RNA-seq-based patient group		Pathology-based patient group	
		TLS-high	TLS-low	TLS-present	TLS-absent	TLS-high	TLS-low	TLS-present	TLS-absent
Total		41	52	38	62	92	93	50	138
Gender	Female (%)	14 (34%)	9 (17%)	7 (18%)	19 (31%)	4 (10%)	7 (20%)	1 (6%)	10 (16%)
	Missing	0	0	0	0	51	58	34	76
Age	Mean	63.0	60.1	61.4	61	61.8	64.5	61.5	63.2
	Missing	1	0	0	0	51	58	34	76
Pathological stage	Stage I	2	0	2	2	NA	NA	NA	NA
	Stage II	9	5	6	9	NA	NA	NA	NA
	Stage III	5	9	7	7	NA	NA	NA	NA
	Stage IV	22	33	22	36	19	17	7	30
	Missing	3	5	1	8	73	76	43	108
HPV status	HPV+	7	3	6	4	14	15	6	23
	HPV−	30	49	28	51	11	6	7	12
	Missing	4	0	4	11	67	72	37	103
Treatment	Chemo	NA	NA	NA	NA	4	2	0	6
	IO	NA	NA	NA	NA	12	13	6	21
	Chemo + IO	NA	NA	NA	NA	25	20	10	35
	Missing	NA	NA	NA	NA	51	58	34	76

TLS, tertiary lymphoid structure; TCGA, The Cancer Genome Atlas; HNSCC, head and neck squamous cell carcinoma; RWE, Real-World Evidence; HPV, human papillomavirus; NA, not available.

### TLS visual assessment on H&E WSIs

Slide-level TLS visual assessment on H&E WSIs was performed by Genmab pathologists through Concentriq LS (Proscia, PA, USA), and a second pathologist reviewed these annotations. TLS was defined histologically as an organized follicular-like structure of lymphoid cell aggregation of predominant B cell lineage, with the presence of plasma cells and T cells. The presence of at least one dendritic cell and inclusive or adjacent high endothelial venules were also included as characteristic features of TLSs on H&E images [2,31]. H&E images with predominant necrosis, significant staining, or tissue artifacts and from metastatic lymph node samples were not included for TLS visual assessment. TLS annotations as region of interest markings from a subset of images were generated for visual interpretability. Both the annotating pathologist and the reviewing pathologist were blinded to transcriptomic clustering results and model outputs.

### TLS gene signature calculation and clustering

Bulk RNA-seq from TCGA and the RWE cohort produces a per-sample expression profile, i.e., a vector of normalized expression values across genes (e.g., transcripts per million). Gene set variation analysis (GSVA) [32] is an unsupervised, nonparametric method used in bioinformatics to estimate the activity of biological pathways (gene sets) on a single-sample basis. In this study, GSVA scores were calculated using RNA-seq data for 6 previously published TLS gene signatures, yielding 6 continuous scores per sample that were subsequently used for clustering into TLS-high/low. *K*-means clustering [33] was further applied to the 6 GSVA scores to derive TLS-high and TLS-low clusters. *K* = 2 was chosen to create a binary stratification comparable to pathology-based TLS status. Uniform manifold approximation and projection (UMAP) algorithm [34] was used to visualize the clusters.

### Correlation between pathology annotation and TLS signature

Correlation between pathologist-generated TLS label (TLS-present and TLS-absent) and TLS-gene-signature-based *K*-means clusters (TLS-high and TLS-low) were directly evaluated to calculate the concordance score [*N*_(TLS-present==TLS-high)_ + *N*_(TLS-absent==TLS-low)_)/*N*_(Total overlapping samples)_]. Welch’s *t* test [35] was applied to compare TLS GSVA scores between pathologist-generated TLS-present and TLS-absent groups for both TCGA and RWE cohorts separately for both indications.

### Image processing

Slideflow [36] package was used for image processing. Tiles were extracted from the WSIs at 20× magnification and 299 in pixels. During the process, poor-quality tiles including background tiles, blurry tiles, or tiles with high whitespace content (>60%), were discarded. Stain normalization was applied using Reinhard algorithm [37].

### Feature extraction and multiple-instance learning

Five different foundation models, including RETCCL, Histossl, CTransPath, PLIP, and Virchow, as well as an ImageNet-trained ResNet50, were used to extract features from image tiles derived from each WSI (Table 3).

**Table 3.** Summary of the evaluated pathology foundation models

Model	Model size (parameters)	Number of images	Number of tiles (millions)	Training algorithm	Image source
CTransPath	28 million	32,220	16	SRCL	TCGA, PAIP
Histossl	86 million	6,093	43	iBOT	TCGA
Virchow	631 million	1,488,000	2,000	DINOv2	Memorial Sloan Kettering Cancer Center
PLIP	Not specified	208,414	Not specified	Contrastive Learning	Twitter, LAION
RETCCL	Not specified	Not specified	Not specified	Contrastive Learning	Custom histopathological datasets
ResNet50	25.6 million	1,280,000	Not specified	ResNet50	ImageNet

TCGA, The Cancer Genome Atlas.

The dimensions of the image features ranged from 512 to 2,560. The multibranch-clustering-constrained attention multiple-instance learning (CLAM_MB [38]) model was used to aggregate and evaluate the effectiveness of the image features. To optimize model performance, a grid search was conducted over a set of hyperparameters, including learning rate, model size, bag size, and batch size. The image set for both indications were split into 75% training and 25% testing strictly at patient level. HNSCC TCGA and RWE images were mixed before splitting into training and testing sets. A 3-fold cross-validation method was applied on the training data to evaluate the performance of each hyperparameter combination. The best combination of hyperparameters were run 3 times to generate 9 data points to have a more accurate estimation. The optimized model was trained on all the training images to predict the outcome in the test data. We utilized several evaluation metrics including accuracy, specificity, recall, area under the curve (AUC), and F1 score to measure the model performance.

### Model interpretation with attention heatmaps

Attention heatmaps [39] were generated using the best foundation model in cross-validation to highlight the most relevant regions in predicting the TLS labels. The heatmaps were then further reviewed by a pathologist to evaluate the pathological relevance of the highlighted regions in TLS identification. TLS pathology annotation of the same images was also generated before pathologists reviewed the heatmaps.

## Results

### Patient demographics

A total of 173 patients with PDAC from TCGA and 288 patients with HNSCC from TCGA (*n* = 100) and the RWE cohort (*n* = 188) with pathology assessment were included in this study. Among these, 163 of 173 patients with PDAC and 278 of 288 patients with HNSCC had matched RNA-seq data. No significant differences were observed between TLS-signature-based TLS-high and TLS-low groups or pathology-based TLS-present and TLS-absent groups in terms of gender, age, or pathological stage. Most of patients with PDAC were classified as stage 2 (Table 1). Similarly, within each HNSCC cohort, no significant differences were detected in patient characteristics, including gender, age, disease stage, human papillomavirus (HPV) status, or treatment history, between TLS groups (signature-based or pathology-based). In addition, demographic characteristics, including gender, age, and disease stage, were comparable between the HNSCC-TCGA and HNSCC-RWE cohorts (Table 2). However, most HNSCC-TCGA patients were immunotherapy (IO) naïve, whereas most of HNSCC-RWE patients had received prior treatment. Furthermore, HPV positivity was lower in the HNSCC-TCGA cohort (11.24%) compared to the HNSCC-RWE cohort (60.4%).

### TLS label generation and evaluation

**Fig. 2.**
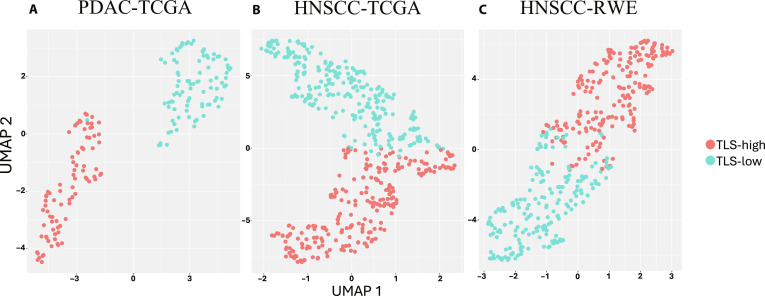
Patient clustering with tertiary lymphoid structure (TLS) signatures in pancreatic ductal adenocarcinoma (PDAC)-The Cancer Genome Atlas (TCGA) (A), head and neck squamous cell carcinoma (HNSCC)-TCGA (B), and HNSCC-Real-World Evidence (RWE) (C). Red cluster represented TLS-high samples, and blue cluster represented TLS-low samples.

We first generated GSVA scores using transcriptomic data from TCGA and the RWE cohort for the 6 published TLS signatures (Table S1). A *K*-means clustering algorithm was applied to classify TLS-high and TLS-low groups (Fig. 2) for PDAC, HNSCC-TCGA, and HNSCC-RWE separately. For PDAC, we identified 84 TLS-high and 89 TLS-low slides, and for HNSCC, we identified 133 TLS-high (41 TCGA + 92 RWE) and 145 TLS-low (52 TCGA + 93 RWE) slides. Separately, pathologists performed a pathological review on H&E images to classify 91 TLS-present and 72 TLS-absent slides for PDAC and 88 TLS-present (38 TCGA + 50 RWE) and 200 TLS-absent (62 TCGA + 138 RWE) for HNSCC. TLS maturity status was not determined because of insufficient clarity from H&E-stained images. Notably, significant variations of TLS histological features between PDAC and HNSCC H&E WSIs are appreciated, such as the size, number of immune cells, cell density, maturation status, and spatial distribution in TME on H&E images. Concordance between pathology-assessed TLS levels and *K*-means clustering was 56.6% for PDAC and 41.0% for HNSCC (54.9% TCGA and 34.06% RWE; Fig. S1).

The lower concordance rate in HNSCC was observed primarily in the RWE cohort, which exhibited greater heterogeneity in the images. To further explore the correlation between pathological review and individual TLS signatures, we conducted Welch’s *t* test to compare TLS signature levels between TLS-present and TLS-absent groups in both PDAC and HNSCC (Fig. S2). In PDAC, TLS-present cases showed consistently higher TLS signature levels (*P* < 0.05) in 5 of 6 different TLS signatures (Fig. S2). In the HNSCC cohort, TLS-present tumors similarly displayed significantly higher TLS signature levels (*P* < 0.05) in 5 of 6 signatures in the HNSCC-RWE cohort. However, none of the comparisons in HNSCC-TCGA reached significance, despite showing similar trends (Fig. S2).

### TLS prediction in PDAC

**Fig. 3.**
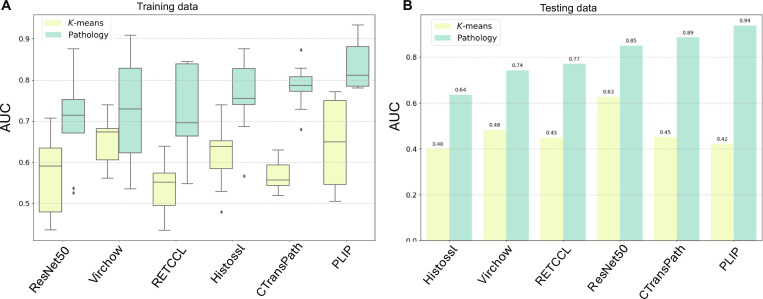
Performance of foundation models in predicting tertiary lymphoid structures (TLSs) in pancreatic ductal adenocarcinoma (PDAC) of (A) cross-validation (3 × 3) of training set and (B) testing set.

The performance of 6 feature extractors—ResNet50, Histossl, RETCCL, CTransPath, PLIP, and Virchow—on predicting both RNA-seq-based and pathology-derived TLS labels in the PDAC cohort is presented in Fig. 3. Overall, all models struggled to predict RNA-seq-based labels, with average AUCs ranging from 0.54 to 0.65 using 3 × 3 cross-validation (Fig. 3A and Table S2). In contrast, all models performed better in predicting pathology labels with AUCs ranging from 0.71 to 0.84 with 3 × 3 cross-validation (Fig. 3A and Table S3). PLIP achieved highest AUC of 0.84 (±0.06), followed by CTransPath with an AUC of 0.78 (±0.06) on the training data (Fig. 3A and Table S3). This trend persisted in the testing data, where all models demonstrated improved performance on pathology labels (AUC: 0.64 to 0.94) compared to *K*-means (AUC: 0.40 to 0.63) (Fig. 3B and Table 4). PLIP exhibited particularly robust performance on the testing data, with an AUC of 0.94 and an F1 score of 0.95 when predicting pathology labels. CTransPath closely followed, achieving an AUC of 0.89 and an F1 score of 0.91. Surprisingly, ImageNet-trained ResNet50 also showed respectable performance, with an AUC of 0.85 and an F1 score of 0.86. The best-performing feature extractor for *K*-means predictions on testing data was ImageNet-pretrained ResNet50, with an AUC of 0.63 and an F1 score of 0.67 (Fig. 3B and Table 4).

**Table 4.** Detailed performance of foundation models in predicting TLSs in PDAC test data

Outcome	Extractor	Accuracy	Specificity	Recall	ROC_AUC	F1 score
TLS_present	PLIP	0.94	0.93	0.95	0.94	0.95
TLS_present	CTransPath	0.89	0.83	0.94	0.89	0.91
TLS_present	ResNet50	0.85	0.86	0.84	0.85	0.86
TLS_present	RETCCL	0.79	0.67	0.88	0.77	0.82
TLS_present	Virchow	0.76	0.64	0.84	0.74	0.8
TLS_present	Histossl	0.67	0.43	0.84	0.64	0.74
*K*-means	ResNet50	0.63	0.56	0.7	0.63	0.67
*K*-means	RETCCL	0.45	0.33	0.57	0.45	0.52
*K*-means	CTransP1ath	0.45	0.38	0.52	0.45	0.5
*K*-means	PLIP	0.42	0.44	0.4	0.42	0.42
*K*-means	Histossl	0.39	0.5	0.3	0.40	0.34
*K*-means	Virchow	0.47	0.67	0.3	0.48	0.37

TLS, tertiary lymphoid structure; PDAC, pancreatic ductal adenocarcinoma; ROC, receiver operating characteristic; AUC, area under the curve.

### TLS prediction in HNSCC

**Fig. 4.**
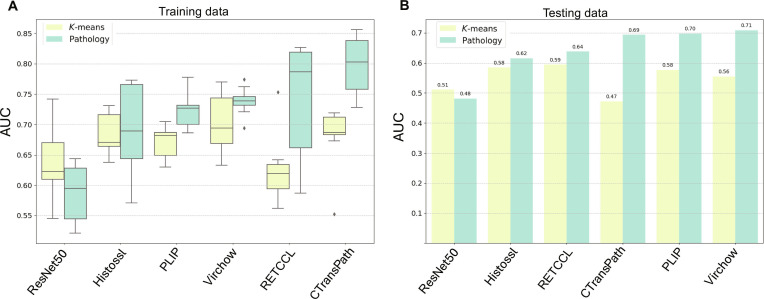
Performance of foundation models in predicting tertiary lymphoid structures (TLSs) in head and neck squamous cell carcinoma (HNSCC) of (A) cross-validation (3 × 3) of training set and (B) testing set.

The same feature extractors were evaluated in the HNSCC cohort for predicting both RNA-seq-based and pathology-derived TLS status (Fig. 4). Similar to PDAC, all models struggled to predict RNA-seq-based labels, with average AUCs ranging from 0.63 to 0.70 in 3 × 3 cross-validation (Fig. 4A and Table S5). Most models again demonstrated better performance on pathology labels, except for ResNet50 [0.59 (±0.05)] (Fig. 4A and Table S5). CTransPath achieved the highest performance, with an AUC of 0.80 (±0.05), followed by Virchow and RETCCL with an AUC of 0.74 (±0.02) and 0.74 (±0.09), respectively (Fig. 4A and Table S5). In testing data, however, all models struggled with both pathology and *K*-means predictions, with Virchow achieving the highest AUC of 0.71 and an F1 score of 0.59 for pathology label predictions (Fig. 4B and Table 5). Notably, ResNet50 performed poorly on the HNSCC pathology label with an AUC of 0.48 and an F1 score of 0.35, a marked contrast to its robust performance in PDAC (Fig. 4B and Table 5). The significant drop in pathology label prediction accuracy in HNSCC compared to PDAC may be attributable to a more complex TME, greater heterogeneity in tumor location based on internal pathology assessment and previous publications [40,41], and the mixed dataset from TCGA and the RWE cohort.

**Table 5.** Detailed performance of foundation models in predicting TLSs in HNSCC test data

Outcome	Extractor	Accuracy	Specificity	Recall	ROC_AUC	F1 score
TLS_present	Virchow	0.72	0.76	0.68	0.71	0.59
TLS_present	PLIP	0.7	0.71	0.68	0.70	0.58
TLS_present	CTransPath	0.72	0.76	0.63	0.69	0.57
TLS_present	RETCCL	0.64	0.64	0.63	0.64	0.51
TLS_present	Histossl	0.61	0.6	0.63	0.62	0.49
TLS_present	ResNet50	0.48	0.49	0.47	0.48	0.35
*K*-means	Virchow	0.56	0.41	0.56	0.56	0.58
*K*-means	RETCCL	0.6	0.48	0.71	0.59	0.66
*K*-means	PLIP	0.59	0.45	0.71	0.58	0.65
*K*-means	Histossl	0.59	0.55	0.62	0.58	0.62
*K*-means	CTransPath	0.48	0.41	0.53	0.47	0.52
*K*-means	ResNet50	0.51	0.55	0.47	0.51	0.51

TLS, tertiary lymphoid structure; HNSCC, head and neck squamous cell carcinoma; ROC, receiver operating characteristic; AUC, area under the curve.

### Model interpretation

**Fig. 5.**
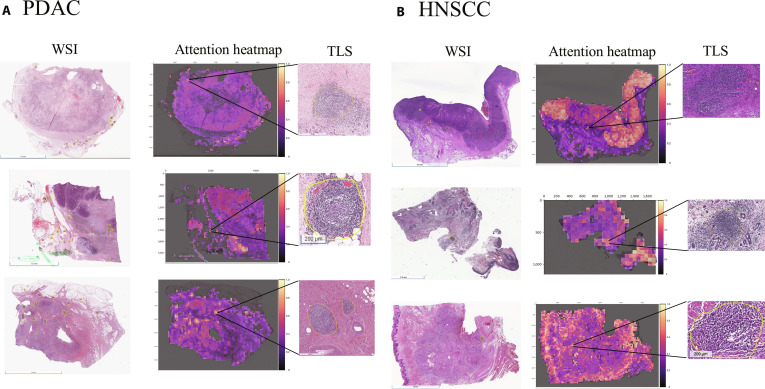
Attention maps of pancreatic ductal adenocarcinoma (PDAC) (A) and head and neck squamous cell carcinoma (HNSCC) (B) slides with tertiary lymphoid structures (TLSs). High-attention regions selected are representative examples to illustrate typical true-positive TLSs. Attention maps are shown for qualitative interpretation only.

To better understand how the model detected the TLSs and explore the disparities in model performance, pathology annotation and attention heatmaps were generated for both PDAC and HNSCC cohorts (Fig. 5). Attention heatmaps highlight regions of histopathology slides that contribute most to the model prediction, offering crucial interpretability in digital pathology by allowing pathologists to assess whether the AI is focusing on biologically and clinically relevant tissue features. Attention heatmaps generated by the CLAM on PLIP features for pathology-based TLS status in PDAC were shown in Fig. 5A (middle). Qualitatively, high-attention regions frequently overlapped with areas a pathologist considered compatible with TLS morphology in representative PDAC cases (Fig. 5A, right). In contrast, although attention heatmaps (Fig. 5B, middle) generated with Virchow-extracted features for HNSCC could still represent TLSs (Fig. 5B, right), they often struggled to focus on TLS regions only. Pathological evaluation indicated that the high-attention tiles identified by the model in HNSCC were influenced by preexisting lymph nodes and non-TLS immune cell infiltration, which may exhibit visual similarities to TLSs. However, we did not perform a formal quantitative overlap analysis; therefore, attention maps are presented as illustrative examples to support interpretability rather than as a validated performance metric.

## Discussion

Self-supervised learning has significantly advanced computational pathology by enabling the training of foundation models on large pathology image datasets. The increasing availability of publicly released models by both academic and private institutions is fostering innovation in the next generation of predictive pathology tools. Foundation models, particularly in weakly supervised algorithms, have demonstrated superior performance and generalizability compared to traditional supervised methods. These models have been instrumental in cancer research, facilitating tumor diagnosis, biomarker prediction, and prognostic assessments [42,43]. Since 2022, foundation models have been integrated into weakly supervised pipelines, leading to improved diagnostic accuracy and broader applicability. As more foundation models are trained, benchmarking studies on clinically relevant tasks have also become available. Campanella et al. [42] benchmarked 8 foundation models on 9 disease detection and 11 biomarker prediction tasks, finding that while newer models generally outperformed ImageNet-pretrained encoders and CTransPath, their performance varied by task and was not significantly impacted by model size. Similarly, Neidlinger et al. [43] demonstrated that model performance varied by task, with the quality and cleanliness of training data outweighing data volume or the training algorithm.

In this study, we evaluated the utility of the pathology foundation models for a unique task, which is to detect TLS presence, defined by morphological features or transcriptomic gene signatures in PDAC and HNSCC. It appears to show low concordance between transcriptomic-signature-based TLS grouping and morphology-based TLS status, and this should not be taken as evidence of a true inverse biological relationship. As a matter of fact, the 2 labels actually are not completely interchangeable: Pathology identifies spatially organized lymphoid aggregates visible in the section, whereas bulk RNA TLS signatures can reflect diffuse immune activation, tumor purity, and sampling/admixture and may be influenced by lymphoid structures that are not TLSs in the analyzed slide. These effects are likely amplified in HNSCC due to greater microenvironmental heterogeneity and lymphoid confounders, leading to systematic discordance between morphology and transcriptomic signatures.

As previously shown, detecting TLSs via computational methods typically requires extensive annotation and segmentation efforts [21–24]. Here, we demonstrate that pathology foundation models can serve as an effective and out-of-the-box alternative for detecting morphologically defined TLSs in tumor types with less complex TME. Consistent with Campanella et al. [42] and Neidlinger et al. [43], we also found that model performance varies by difficulty of the task, while model size was not a significant factor. In general, pathology foundation models outperformed the ImageNet-pretrained ResNet50 in both the PDAC and HNSCC cohorts when predicting morphology-based TLS status. Although the foundation models consistently outperformed ResNet50 in HNSCC slide images, they all achieved lower mean AUC scores compared to their performance on PDAC tumors. This disparity is likely due to the heterogeneous TME of HNSCC, which may cause the foundation models to struggle in accurately identifying specific factors among multiple complex features. For example, preexisting lymph nodes were frequently present in the HNSCC TME and may have affected the model’s performance.

The largest foundation model evaluated, Virchow, did not demonstrate superior performance over smaller models, despite narrowly achieving the highest performance on the HNSCC testing data. This aligns with previous findings that model size does not necessarily correlate with performance.

When predicting pathology-based TLSs in PDAC, ResNet50 achieved a comparable AUC to most pathology foundation models in the training set and ranked third in the testing set. Interestingly, in the HNSCC cohort, the gap between pathology foundation models and ResNet50 widened, with foundation models consistently outperforming ResNet50 in predicting pathology-based TLSs. These findings suggest that while pathology-image-pretrained models still have room for improvement, they provide the most value in pathologically challenging tasks.

Architecture and pretraining differences may also contribute to model performance. Beyond disease-specific factors (e.g., greater immune heterogeneity and lymphoid confounders in HNSCC), performance differences across encoders may reflect differences in architecture, scale, pretraining objective, and data domain (Table 3). CTransPath (CNN + modified transformer; semantically relevant contrastive learning [SRCL] on TCGA/PAIP) may benefit from combining local texture sensitivity with broader contextual modeling. Histossl (ViT; iBOT/DINOv2-style self-supervision on TCGA) and Virchow (ViT-Huge; DINOv2 on a large institutional pathology corpus) may gain from pathology-native pretraining and, for Virchow, substantially larger capacity and training diversity. In contrast, PLIP (contrastive language–image pretraining-style vision–language contrastive pretraining on Twitter/LAION) may offer robust general representations but introduces domain shift relative to clinical H&E, while RETCCL (ResNet50; clustering-guided contrastive learning on histopathology datasets) emphasizes retrieval-oriented, discriminative features. Because we use frozen encoders within a shared multiple instance learning framework, these differences likely reflect representation transferability rather than task-specific finetuning.

Contrary to pathology-based label prediction, all pathology models performed poorly and did not outperform ResNet50 in identifying signature-based TLS status. This could suggest that while the foundation models excel in H&E image recognition capabilities, they struggle in RNA-based biomarker predictions, highlighting a potential limitation in their versatility across modalities. This may be partly due to the fact that TLS gene signatures are not necessarily TLS-specific and may not directly correlate with morphological features visible on H&E-stained slides. Given that current pathology foundation models largely rely on morphological features, incorporating omics data into their training could improve predictive performance, especially when target labels are defined by molecular or transcriptomic signatures [44]. The other limitation in signature-based TLS status is that *K*-means clustering was used to generate the label in this study, while unsupervised binarization may introduce label uncertainty, and that future work will evaluate cluster stability and alternative thresholding strategies.

Overall, model performance varied by task complexity rather than size, aligning with recent benchmarking findings. One limitation of this study is that only 5 pathology foundation models were evaluated, each pretrained using different image sizes and algorithms. This selection was not intended to provide an exhaustive benchmark of all available models. Rather, the aim was to illustrate the practical utility and inherent limitations of representative models within the context of this specific application. Another limitation is that attention visualizations were not compared systematically across all foundation models; future work will evaluate whether lower-performing models exhibit distinct or less specific attention patterns under standardized region of interest selection.

## Conclusion

This study demonstrated that pathology foundation models effectively detected TLS presence in PDAC and HNSCC, outperforming ImageNet-pretrained ResNet50 in morphology-based predictions. However, foundation models struggled with transcriptomic-signature-based TLS prediction, revealing cross-modal generalization challenges, and performed worse in heterogeneous HNSCC microenvironments, highlighting limitations in complex pathological contexts. Despite these challenges, pathology foundation models show strong potential as the backbone of automated digital pathology pipelines for task-specific applications. The widespread availability and standardization of H&E-stained images make them a compelling source for model deployment and scaling, particularly in biomarker discovery and patient stratification. Continued refinement will be essential to enhance their versatility and robustness across modalities and tumor heterogeneity.

## Ethical Approval

This study was conducted on deidentified health information subject to an institutional review board exempt determination (Advarra Pro00072742) and did not involve human subject research.

## Data Availability

TCGA data were accessible from https://portal.gdc.cancer.gov/. The commercial cohort consisted of deidentified data collected in a real-world healthcare setting that are subject to controlled access for privacy and proprietary reasons. When possible, derived data supporting the findings of this study have been made available within the paper and its supplementary figures/tables. Restrictions apply to the availability of additional data, which were used under license for this study. Portions of the code (or a runnable subset without proprietary components) may be shared upon reasonable request, subject to institutional approval and appropriate agreements. Requests should be directed to the corresponding author.
